# Influence of age and antiepileptic comedication on lacosamide concentrations in children with epilepsy: A retrospective cohort study

**DOI:** 10.1371/journal.pone.0333030

**Published:** 2025-10-16

**Authors:** Zhaosong Du, Hui Peng, Jun Wang, Maochang Liu, Gang Nie, Ying Li

**Affiliations:** 1 Department of Pharmacy, Wuhan Children’s Hospital (Wuhan Maternal and Child Healthcare Hospital), Tongji Medical College, Huazhong University of Science & Technology, Wuhan, Hubei, China; 2 Division of Gastroenterology, Union Hospital, Tongji Medical College, Huazhong University of Science & Technology, Wuhan, Hubei, China; INNN: Instituto Nacional de Neurologia y Neurocirugia Manuel Velasco Suarez, MEXICO

## Abstract

**Object:**

To explore the influencing factors of lacosamide serum concentration in children with epilepsy, and to provide evidence-based guidance for individualized dosing strategies.

**Methods:**

Clinical data of pediatric epilepsy patients treated with lacosamide from September 2021 to January 2025 were retrospectively analyzed. Participants were stratified by age into ≤6, 6–12, and >12 years groups. Non-parametric tests were employed to compare differences in daily dose, weight-adjusted daily dose, serum concentration, and concentration-to-dose ratio (CDR) across sex, age, and concomitant antiepileptic drugs (AEDs) groups. Multiple linear regression analysis was conducted to identify independent factors influencing serum concentration.

**Results:**

The study enrolled 438 patients (boys: 261, 59.59%; girls: 177, 40.41%), with age distribution as follows: ≤ 6 years (n = 85, 19.41%), 6–12 years (n = 294, 67.12%), and >12 years (n = 59, 13.47%). The > 12 years group exhibited significantly higher daily dose, weight-adjusted daily dose, and CDR compared to younger cohorts. Concomitant use of non-hepatic enzyme-inducing AEDs resulted in elevated serum concentration and CDR relative to lacosamide monotherapy. Regression analysis identified body weight, weight-adjusted daily dose, and coadministration of non-hepatic enzyme-inducing AEDs as independent predictors of serum concentration.

**Conclusion:**

Age and concomitant non-hepatic enzyme-inducing AEDs therapies significantly influence lacosamide exposure, underscoring the necessity for age-based dynamic dose optimization and rigorous evaluation of polypharmacy regimens to achieve precision therapeutics.

## Introduction

Lacosamide (LCM), as a third-generation antiepileptic drug, was approved for marketing in China in 2018 and was approved by the National Medical Products Administration to expand its indications in January 2021, becoming a monotherapy option for patients aged 4 years and above with focal seizures. Evidence-based medicine has indicated that lacosamide has shown good therapeutic effects and tolerance in adult and pediatric epilepsy patients [1–4]. Based on its superior pharmacological properties, many international guidelines for the diagnosis and treatment of epilepsy have included it in the adjunctive treatment of generalized epilepsy and the first-line drug recommendation for focal epilepsy [5,6]. However, at present, there is still a lack of clear consensus on the effective therapeutic window of lacosamide in children with epilepsy, and there are also few studies on the influencing factors of its serum concentration. Especially considering the characteristics of its serum concentration-dependent adverse reactions, this issue has become a key factor restricting the rational use of drugs in clinical practice. A study pooling data from three pivotal double-blind, placebo-controlled trials of adjunctive lacosamide in adults with partial-onset seizures indicates that common drug-associated treatment-emergent adverse events generally appeared to be dose-related [7]. Another retrospective multicenter cohort study examined the safety of intravenous lacosamide use in 686 children and 28 neonates, showing there was a twofold increase in the risk of rash in the higher dose cohort when compared to the recommended initial dose [4]. It is worth noting that due to the unique physiological development characteristics and pharmacokinetic differences of the child group, coupled with the significant ethical challenges and operational difficulties in clinical research involving special populations such as newborns and infants, the pharmacokinetic characteristics of LCM in children have not been fully clarified.

Based on the above current situation, this study systematically collected the medication data of children with epilepsy by conducting therapeutic drug monitoring (TDM), aiming to explore the key factors affecting the serum concentration of lacosamide and provide an evidence-based basis for establishing individualized drug administration regimens for children.

## Materials and methods

### Patients’ information

This is a retrospective study. The basic data of children diagnosed with “epilepsy” in the neurology outpatient department from September 2021 to January 2025 were collected, including data such as age, gender, weight, medication regimens, serum concentrations, and the dosage form of lacosamide. The exclusion criteria for the child patients are: (1) those with any underlying diseases; (2) those who have not undergone serum concentration monitoring of lacosamide; (3) those with missing data and information; (4) those with abnormal liver and kidney functions. The data used in this study were extracted from the scientific research data platform of Wuhan Children’s Hospital. The protocol was reviewed and approved by the Medical Ethics Committee of Wuhan Children’s Hospital. Due to the nature of this retrospective study, the requirement for informed consent has been waived. Ethics Batch Number: 2025R033-E01.

### Methods

This study established an analytical method for the detection of serum lacosamide concentration by solid-phase extraction-high performance liquid chromatography (HPLC) [8]. Blood sampling for steady-state drug concentration measurement was performed after a minimum of 30 days following treatment initiation. This interval exceeds the 5 elimination half-life of lacosamide (approximately 13 hours), ensuring that steady-state conditions were achieved for all patients prior to sampling [9,10]. 3 mL of venous blood samples were collected on an empty stomach the next morning. The sample was first collected by centrifugation at 5000 r/min for 2 minutes. Precisely measure 0.5 mL of the serum sample and treat it with an ODS C18 solid-phase extraction column (with 500 mg packing material and 3 mL column volume). After the adsorbent was fully impregnated, it was naturally drained. The column was washed successively with 1 mL of 0.9% sodium chloride injection. Discarding the washing solution, the target substance was eluted with 1 mL of 70% methanol solution. The eluent was analyzed by high-performance liquid chromatography.

Chromatographic analysis was conducted using the Agilent 1260 high-performance liquid chromatography system, and the chromatographic conditions were optimized as follows: The SinoChrom ODS-BP C18 chromatographic column (4.6 mm × 250 mm, 10 μm) was used as the separation medium. The mobile phase was a methanol-water system (40:60, V/V), the flow rate was 0.8 mL·min ⁻ ¹, the detection wavelength was 215 nm, and the column temperature was maintained at 30°C. Methodological validation shows that: The detection limit (LOD) of this method was 0.231 mg·L ⁻ ¹, with a quantitative range of 0.841–26.905 mg·L ⁻ ¹ (the correlation coefficient of the standard curve r = 0.999). The average recovery rates of low, medium, and high concentration quality control samples were all ≥ 98%, and the relative standard deviation (RSD) between the intra-day precision and the inter-day precision was all ≤ 10%. Experimental data showed that this method has good sensitivity, accuracy, and repeatability, and is suitable for the monitoring requirements of clinical therapeutic drugs.

The daily dose of LCM was adjusted based on the patient’s body weight, calculated as the daily dose divided by the child’s weight. Due to large inter-individual variances, the LCM serum concentration at steady-state was adjusted by body weight and dosage of each patient and expressed as concentration to dose ratio of LCM (CDR, (μg·ml^-1^)/(mg·kg^-1^)). The CDR = LCM serum concentration/LCM adjusted daily dose.

In the analysis of the combined use of antiepileptic drugs (AEDs) in LCM, oxcarbazepine, lamotrigine, and clobazam were classified as hepatic enzyme-inducing AEDs. Valproic acid, topiramate, levetiracetam, midazolam, perampanel, clonazepam, and zonisamide were classified as non-hepatic enzyme-inducing AEDs.

### Statistical analysis

Data analyses were carried out using R 4.4.2 software. The normality test was conducted using the graphical method and the Shapiro-Wilk method. The measurement data that did not conform to the normal distribution were expressed as the median (interquartile range) [*M(P*_*25*_*, P*_*75*_*)*]. The Wilcoxon rank sum test was used to compare two groups, and the Kruskal-Wallis *H* test was used to compare multiple groups. If the differences among multiple groups were statistically significant, the post hoc analysis was conducted using the Dunn test. Counting data was expressed as percentages (%). The influencing factors of lacosamide serum concentration were analyzed by multiple linear regression. The variable age group was categorized as ≤6 years (reference group), 6–12 years, and >12 years. The variable gender was categorized as boy (reference group) and girl. The variable concomitant AEDs were categorized as monotherapy (reference group), enzyme inducers, and non-enzyme inducers. *P* values <0.05 indicate that the difference was statistically significant.

## Results

### Demographic characteristics

A total of 438 children with epilepsy were included in this study, with a median age of 8.53 years (6.62–10.643 years), among whom were ≤6 years group, 6–12 years group and >12 years group children accounted for 19.41% (85 cases), 67.12% (294 cases) and 13.47% (59 cases) respectively. The proportion of boys was 59.59% (261 cases), and that of girls was 40.41% (177 cases). The median body weight was 28 kg (22–38.75 kg). Regarding the use of lacosamide, the median daily dose of lacosamide was 120 mg·d ⁻ ¹ (100–150 mg·d ⁻ ¹), and the adjusted daily dose (adjusted for body weight) was 4.428 mg·kg ⁻ ¹ (3.486–5.36 mg·kg ⁻ ¹). Median serum concentration was 3.7 mg·ml^-1^ (2.763–5.077 mg·ml^-1^), the CDR was 0.8745 (0.663–1.1509) (μg·ml ⁻ ¹)/(mg·kg ⁻ ¹). The dosage form was mainly tablets (82.65%). Regarding the combined use of AEDs, 68.72% (301 cases) were treated with lacosamide monotherapy. The proportions of lacosamide combined with hepatic enzyme-inducing AEDs and non-hepatic enzyme-inducing AEDs were 6.62% (29 cases) and 24.66% (108 cases), respectively (Table 1).

**Table 1:** Demographic characteristics and clinical information of the children with epilepsy.

Variables	Enrolled patients (n = 438)
Age [*M*(*P*_25_, *P*_75_), year]	8.53(6.62,10.64)
Age group [n(%)]	
≤ 6 years	85(19.41)
6–12 years	294(67.12)
> 12 years	59(13.47)
Gender [n(%)]	
Boy	261(59.59)
Girl	177(40.41)
Body weight [*M*(*P*_25_, *P*_75_), kg}	28(22,38.75)
Daily dose [*M*(*P*_25_, *P*_75_), mg·d ⁻ ¹]	120(100,150)
Adjusted daily dose [*M*(*P*_25_, *P*_75_), mg·kg ⁻ ¹]	4.43(3.49,5.36)
Serum concentration [*M*(*P*_25_, *P*_75_),μg·ml ⁻ ¹]	3.7(2.76,5.08)
CDR [*M*(*P*_25_, *P*_75_),(μg·ml ⁻ ¹)/(mg·kg ⁻ ¹)]	0.87(0.66,1.15)
Dosage form [n(%)]	
Tablets	362(82.65)
Oral solution	76(17.35)
Concomitant AEDs [n(%)]	
Monotherapy	301(68.72)
Enzyme inducers	29(6.62)
Non-enzyme inducers	108(24.66)

*CDR*: concentration-to-dose ratio; *AEDs*: antiepileptic drugs.

### The influence of age, gender, and concomitant AEDs on the lacosamide serum concentration

The effects of age, gender, and concomitant AEDs on the serum concentration of lacosamide are shown in Table 2. After grouping the children by age, there were significant differences in daily dose, adjusted daily dose, and CDR among different age groups (*P* < 0.01). The daily dose in the > 12 years group (200 mg·d ⁻ ¹) was significantly higher than that in the ≤ 6-years group (100 mg·d ⁻ ¹) and the 6−12 years group (125 mg·d ⁻ ¹) (*P* < 0.01), but the adjust daily dose (3.41 mg·kg ⁻ ¹) was significantly lower than that of the other two groups (*P* < 0.01). The > 12 years group had the highest CDR (1.19(μg·ml^-1^)/(mg·kg^-1^)), which was significantly higher than that of the ≤ 6-years group (0.71(μg·ml^-1^)/(mg·kg^-1^)) and the 6–12-years group (0.859(μg·ml^-1^)/(mg·kg^-1^)) (*P* < 0.01).

**Table 2:** Comparison of the daily dose, adjusted daily dose, serum concentration, and CDR of lacosamide among different groups.

Variable	N	Daily dose [*M*(*P*_25_, *P*_75_), mg·d^-1^]	Adjusted daily dose [*M*(*P*_25_, *P*_75_), mg·kg^-1^]	Serum concentration [*M*(*P*_25_, *P*_75_),μg·ml^-1^]	CDR [*M*(*P*_25_, *P*_75_), (μg·ml^-1^)/(mg·kg^-1^)]
Age group					
≤ 6 years	85	100(60,100)	5(4,6.36)	3.39(2.62,4.87)	0.71(0.56,0.94)
6–12 years	294	125(100,150)*	4.47(3.7,5.26)*	3.72(2.78,5.03)	0.86(0.68,1.11)*
> 12 years	59	200(100,250)*†	3.41(2.5,4.84)*†	3.91(3.04,5.45)	1.19(0.97,1.45)*†
*Χ*^2^		85.15	23.55	3.14	45.18
*P* value		<0.01	<0.01	0.21	<0.01
Gender					
Boy	261	120(100,150)	4.29(3.33,5.26)	3.71(2.82,5.15)	0.92(0.68,1.16)
Girl	177	120(100,150)	4.65(3.75,5.45)	3.67(2.72,4.91)	0.86(0.63,1.12)
W		23454	21117	23469	25139
*P*		0.78	0.13	0.78	0.12
Concomitant AEDs					
Monotherapy	301	120(110,150)	4.36(3.57,5.26)	3.56(2.71,4.9)	0.85(0.63,1.12)
Enzyme inducers	29	100(70,140)*	4.4(2.5,6.36)	3.37(2.76,4.11)	0.83(0.6,1.07)
Non-enzyme inducers	108	120(100,150)†	4.57(3.33,5.58)	4.27(3.17,5.51)*	0.96(0.74,1.23)*
*Χ*^2^		7.28	0.16	9.88	9.61
*P*		0.03	0.92	<0.01	<0.01

*CDR*: concentration-to-dose ratio; *AEDs*: antiepileptic drugs; *means the difference was significant when compared with the ≤ 6 years group or monotherapy group; † means the difference was significant when compared with the 6–12 years group or enzyme inducers group.

The daily dose in the combined with hepatic enzyme-inducing AEDs group (100 mg·d^-1^) was significantly lower than that in the monotherapy group (120 mg·d^-1^) (*P* = 0.026); The serum concentration (4.27 μg·ml^-1^) and CDR (0.959(μg·ml^-1^)/(mg·kg^-1^)) in the combined with non-hepatic enzyme-inducing AEDs group were significantly higher than those in the monotherapy group (*P* < 0.01).

In the gender grouping, there were no significant differences in daily dose, adjusted daily dose, serum concentration, and CDR between different genders (*P* > 0.05).

### Multiple linear regression analysis of influencing factors of lacosamide serum concentration

The possible influencing factors of lacosamide were included in the covariates for multiple linear regression analysis. It was found that the adjusted daily dose (β = 0.495, *P* < 0.01) and body weight (β = 0.026, *P* < 0.01) were significantly positively correlated with the serum concentration. In the concomitant AEDs, the serum concentration in the group of lacosamide combined with non-hepatic enzyme-inducing AEDs was significantly higher than that in the group of lacosamide monotherapy (β = 0.47, *P* < 0.01). The influence of age segmentation and gender on serum concentration was not statistically significant (*P* > 0.05) (Table 3).

**Table 3:** Multiple linear regression analyses for the influence factors of LCM concentration.

Variable	β	Standard error	t-Value	*P* value
Intercept	0.75	0.46	1.63	0.1
Age group				
≤ 6 years(refer)				
6–12 years	0.09	0.23	0.41	0.68
> 12 years	0.28	0.38	0.75	0.45
Gender				
Boy(refer)				
Girl	0.01	0.15	0.03	0.97
Body weight	0.03	0.01	2.88	<0.01
Adjust daily dose	0.50	0.05	9.25	<0.01
Concomitant AEDs				
Monotherapy(refer)				
Enzyme inducers	−0.19	0.31	−0.6	0.55
Non-enzyme inducers	0.47	0.18	2.68	<0.01

*AEDs*: antiepileptic drugs; ≤ 6 years, boy, and monotherapy were set as the reference group.

### The influence of valproic acid on the lacosamide serum concentration

In order to further investigate the effect of VPA on the lacosamide serum concentration, children with epilepsy were divided into the LCM monotherapy group and the LCM concomitant VPA group. The daily dose, adjusted daily dose, serum concentration, and CDR were evaluated between the two groups. Analysis results indicate that serum concentrations and the CDR were significantly higher in the group receiving LCM in combination with VPA (Table 4).

**Table 4:** The influence of valproic acid on the lacosamide serum concentration.

Variable	N	Daily dose [*M*(*P*_25_, *P*_75_), mg·d^-1^]	Adjusted daily dose [*M*(*P*_25_, *P*_75_), mg·kg^-1^]	Serum concentration [*M*(*P*_25_, *P*_75_),μg·ml^-1^]	CDR [*M*(*P*_25_, *P*_75_), (μg·ml^-1^)/(mg·kg^-1^)]
LCM	301	120(100,150)	4.36(3.57,5.26)	3.56(2.71,4.9)	0.85(0.63,1.12)
LCM + VPA	29	100(100,120)	4.76(3.33,5.36)	5.05(3.41,5.75)	1.1(0.82,1.48)
*W*		5267.5	4163.5	3045	2917.5
*P*		0.06	0.68	<0.01	<0.01

*LCM*: lacosamide; *VPA*: valproic acid; *CDR*: concentration-to-dose ratio.

## Discussion

There have been numerous studies on the influencing factors of lacosamide serum concentration, however, most studies focus on the adult population, and the results were not consistent. This study provided comprehensive insights into the influencing factors of LCM serum concentration and introduced a multivariate linear regression model to assess the independent influencing factors. Importantly, we found the serum concentration of LCM significantly higher in children treated with LCM concomitant with VPA.

In this study, we analyzed the basic conditions and the medication information of lacosamide of 438 children with epilepsy. The median serum concentration of lacosamide in the children was 3.7 μg·ml^-1^. Previous studies have shown that differences in exposure levels of lacosamide can significantly affect its clinical efficacy. A retrospective study involving 72 children with epilepsy aimed at exploring the concentration reference range suitable for Chinese children with epilepsy showed that when the serum concentration was 2.0–7.0 μg·ml ⁻ ¹, the epileptic seizures of 71.4% of the children could be effectively controlled [11]. In another retrospective analysis involving 44 patients with epilepsy, the 10–20 mg/L serum concentration range clearly increases toxicity in patients treated with LCM, showing a correlation between adverse events and total LCM daily dose or LCM serum concentration [12]. Therefore, conducting thorough research on the factors influencing the serum concentration of lacosamide is essential for enhancing its rational clinical application.

Our results revealed that the serum concentration of lacosamide was lower than in previous research. For children, the adverse reactions caused by lacosamide were significantly associated with dosage [4,7]. To avoid the occurrence of adverse reactions, clinicians tend to start the treatment with a low dose. This might result in a relatively low serum concentration of lacosamide in our study. The metabolism and elimination of lacosamide were influenced by multiple factors [13], literature reports that its effective serum concentration for treating children with epilepsy was inconsistent [12,14]. Furthermore, a study investigating the efficacy and tolerability of lacosamide in a pediatric population with epilepsy showed that no statistically significant association was found between LCM serum levels and the clinical response [15]. To date, the recommended reference ranges have varied from 2.2 to 20 mg/L, and data on the LCM reference range are not well established [16,17]. Thus, the serum concentration of LCM in this study remained within a reasonable range.

This study found that the CDR of lacosamide in the > 12 years group was significantly higher than that in the ≤ 6 years and 6–12 years groups. Similar to the results of this study, A retrospective study indicated that LCM trough serum concentrations in children aged 6–12 years and those younger than 6 years were significantly lower (by 21% to 38%) compared to adults aged 18–60 years [18]. Another retrospective study that collected 124 children and adolescents with epilepsy from the therapeutic drug monitoring databases of the Epilepsy centers in Norway and Denmark also found that the adjusted serum concentration of lacosamide was significantly higher in the higher age group (13–17 years group) than in the other two groups (< 6 years group and 6–12 years group) [19]. The influence of age on the serum concentration of lacosamide may be due to the characteristics of children’s growth and development affecting the metabolic process of lacosamide in the body. Previous studies have also shown that antiepileptic drugs have a high clearance rate and a short elimination half-life in younger children [20]. This also led to the adjusted daily dose in this study being significantly higher in the lower age group than in the higher age group.

The influence of gender on the serum concentration of lacosamide is not consistent in adult and child groups. In the adult population, the concentration of lacosamide in females was significantly higher than that in males [21], while in the child population, gender did not affect the serum concentration of lacosamide [15,18]. It was reported that among children of different genders, the main difference lies in the UGT enzyme system, while the difference in the CYP enzyme system was relatively small [22,23]. In vivo and in vitro studies have confirmed that CYP2C19 and CYP2C9 enzymes in the CYP450 enzyme system play a major role in the metabolism of lacosamide [24–26], which may be the main reason why there was no difference in the serum concentration of lacosamide between different genders in the child population. Furthermore, in a clinical study involving 565 individuals with epilepsy, researchers found that body fat contributed to a difference of ~8%, as after serum concentration normalization by body weight, no differences were observed between females and males [27]. Our study also found that there was no significant difference in the serum concentration of lacosamide and the CDR between different gender groups, and multiple regression analysis showed that body weight was positively correlated with serum concentration, which was consistent with previous studies.

Lacosamide is often used as an adjunctive drug to treat epilepsy in children; exploring the interaction between lacosamide and other drugs has important reference significance for the rational clinical use. A study from Norway showed that the combination of antiepileptic enzyme-induced medicines can reduce the serum concentration of lacosamide by 28% [28]. The results of a statistical analysis of 3,154 blood samples from 973 patients using the generalized estimating equation model showed that the combined use of enzyme inducers (carbamazepine, phenytoin sodium, primidone, phenobarbital, ethosuximide) could reduce the serum concentration of lacosamide by 30%−40%, while the combined use of zonisamide would increase the serum concentration of lacosamide by 10%, and the influence was more significant among the children’s group [18]. All the above studies have shown that the combined use of other antiepileptic drugs can significantly affect the serum concentration of lacosamide in children with epilepsy. In this study, we found that the serum concentration of lacosamide in the lacosamide combined with non-hepatic enzyme-inducing AEDs group (i.e., lacosamide combined with valproic acid, topiramate, levetiracetam, midazolam, perampanel, clonazepam, zonisamide) was significantly higher than that in the lacosamide monotherapy group and the lacosamide combined with hepatic enzyme-inducing AEDs group (i.e., lacosamide combined with oxcarbazepine, lamotrigine, clobazam). After further analysis, our study found that the combined use of valproic acid could significantly increase the serum concentration of lacosamide. Previous research has indicated valproic acid may increase the serum concentrations of variety drugs because of its inhibiting activity [29]. Valproic acid was reported to be a broad-spectrum hepatic drug enzyme inhibitor that can inhibit CYP2C19 and CYP2C9, which metabolize lacosamide. In contrast, oxcarbazepine has only a mild enzyme-inducing effect on CYP2C19 [30]. This may be the main reason why the serum concentration of lacosamide in the group of lacosamide combined with non-hepatic drug enzyme inducers in this study was significantly higher than that in the other two groups.

## Limitation

There are some limitations in this study. The samples of the children patients collected in this study were all from a single center, which might cause a certain bias in the research results. Secondly, this study did not collect data on the types of epilepsy in the children and failed to evaluate the pharmacokinetic differences of lacosamide among children with different types of epilepsy. Finally, the polymorphism of the CYP2C19 gene may also have an impact on the serum concentration of lacosamide, but this study failed to conduct an in-depth exploration of it. However, at present, there are relatively few studies on the influencing factors of lacosamide serum concentration in children with epilepsy. The research results of this article still have important reference significance for rational clinical drug use.

## Conclusion

In summary, age, accompanying AEDs, and the adjusted daily dose are the primary factors influencing lacosamide serum concentration. In clinical practice, when using lacosamide, the age of the child should be comprehensively considered, and combined antiepileptic drugs should be carefully selected.

## Supporting information

S1 Dataset The dataset for statistical analysis. (XLSX)
